# Metagenomic Analysis of the Fecal Virome in Wild Mammals Hospitalized in Pisa, Italy

**DOI:** 10.3390/vetsci12090820

**Published:** 2025-08-26

**Authors:** Maria Irene Pacini, Mario Forzan, Micaela Sgorbini, Dania Cingottini, Maurizio Mazzei

**Affiliations:** Department of Veterinary Sciences, University of Pisa, 56124 Pisa, Italy; mariairene.pacini@phd.unipi.it (M.I.P.); micaela.sgorbini@unipi.it (M.S.); dania.cingottini@phd.unipi.it (D.C.); maurizio.mazzei@unipi.it (M.M.)

**Keywords:** wildlife virome, metagenomics, One Health

## Abstract

Emerging infectious diseases, particularly those of zoonotic origin, often originating from wildlife reservoirs represent a growing threat to global health. Human-driven environmental changes such as habitat fragmentation, climate change, and urban expansion have intensified interactions at the wildlife–domestic animal–human interface, facilitating cross-species viral transmission. Despite their epidemiological importance, systematic virological surveillance of wildlife remains challenging. In this study, we employed shotgun metagenomic sequencing to characterize the virome of wild animals rescued in the Pisa area and hospitalized at the “Mario Modenato” Veterinary Teaching Hospital (VTH) at the University of Pisa. Fecal samples collected from injured wildlife admitted between September 2020 and September 2021 were analyzed to detect both known and novel viruses. This approach builds upon previous PCR-based investigations of the same biological material, enabling a more comprehensive assessment of viral diversity. We adopted a shotgun approach for analyzing six sample pools—four were positive for at least one viral target—identifying diverse viral families, including *Astroviridae*, *Circoviridae*, *Picornaviridae*, *Adenoviridae*, and *Retroviridae*, in asymptomatic wildlife admitted to a veterinary hospital, highlighting their potential role as reservoirs. Our findings provide insights into the influence of environmental and anthropogenic factors on wildlife virome composition and highlight the value of hospital-based sampling strategies for urban viral surveillance. The results contribute to the development of integrated monitoring and prevention strategies within a One Health framework.

## Simple Summary

Emerging infectious diseases, especially zoonotic diseases, often have their reservoirs in wild animals. Human activities like urbanization and climate change increase contact between wild animals, domestic animals, and humans, raising the risk of virus transmission. In this study, we used metagenomic sequencing to investigate the presence of viruses in fecal samples from wild animals rescued in the Pisa area and treated at the Veterinary Teaching Hospital of the University of Pisa. This method allowed us to detect a wide range of viruses, including a few that had not been identified before in the tested animals, offering a broader approach than using traditional techniques. Our results show how human impact and environmental factors could shape the wildlife virome and support the usefulness of hospital-based wildlife sampling for monitoring viruses in urban settings. This work aligns with the One Health approach, integrating human, animal, and environmental health.

## 1. Introduction

Emerging infectious diseases have recently increased in incidence and geographic distribution and have acquired new host [1]. The rise in epidemic events caused by emerging or re-emerging pathogens poses a challenge to human, animal, and environmental health worldwide [2,3,4]. Wildlife populations are important natural reservoirs for many pathogens, including zoonotic viruses, and act as ecological sentinels for the early identification of possible novel health threats [5]. Around 75% of emerging pathogens described in recent decades have a zoonotic origin, often arising from wild animal hosts [2,6]. Human activities such as habitat fragmentation, climate change, and urban expansion have resulted in increasing contact between wildlife and domestic animals, thereby promoting cross-species transmission and the possible spread of pathogens. Despite their epidemiological relevance, it is difficult to conduct representative and systematic wildlife virological surveillance [7]. Advanced methods, including shotgun metagenomic sequencing, have transformed the study of viral communities in wildlife by enabling the detection of known and novel viruses that could be undetectable using conventional techniques [8,9].

In this study, we characterized the virome of wild animals from the Pisa area by analyzing their fecal samples collected at the “Mario Modenato” Veterinary Teaching Hospital (VTH) of the University of Pisa using shotgun metagenomic sequencing. Since July 2010, the Fauna Defense Service of Tuscany has entrusted the VTH to provide a 24-h emergency service for wild animals found injured within the province of Pisa, and the ethical approval of clinical procedures was implicit in this agreement. The animals included in the study were rescued after being injured due to accidents and brought to the hospital. This sampling strategy, which focuses on hospitalized wildlife, provides a valuable opportunity to investigate viral diversity in an urban environment and assess potential health risks at the interface between wildlife, humans, and domestic animals.

The aim of this study is to understand how environmental and anthropogenic factors influence the virome composition of wildlife. This investigation is an extension of previous research conducted on the same samples [10]. Building on the initial virological findings obtained through PCR-based analyses and taking advantage of the availability of the same biological material, we conducted a more in-depth investigation to identify any viral pathogens that may have been undetected using traditional scientific methods. The obtained information could be essential and provide valuable data to support integrated monitoring and prevention strategies within a One Health framework.

## 2. Materials and Methods

### 2.1. Sampling

The study was conducted in the Tuscany region (Italy), one of the Italian areas with the highest density of wild mammals. According to recent estimates, Tuscany hosts approximately 40% of the national population of roe deer (*Capreolus capreolus*), 45% of fallow deer (*Dama dama*), and 30% of wild boar (*Sus scrofa*).

Within Tuscany, the province of Pisa covers approximately 2450 km^2^, the territory is partly flat (25%) but predominantly hilly and mountainous. Land use is divided into 50% agriculture, 40% forest (mainly *oak woods*), and less than 4% urbanized areas. In recent years, the abandonment of agricultural activities has fostered forest expansion, increasing wildlife habitats even near urban centers. The sampling area was classified as previously described [10].

Sampling was carried out on wild mammals hospitalized at the VTH of the University of Pisa, as part of an agreement with the Tuscany Region active since 2010 (“Agreement for the coordinated rescue of wild fauna in distress”). The samples were derived from a total of 47 rescued wild mammals admitted to the VHT between September 2020 and September 2021. The primary cause of admission was road traffic collisions, followed by intraspecific aggression and debilitation. At the time of admission, none of the animals exhibited clinical signs compatible with infectious diseases (Appendix A).

The animals were sampled and categorized according to species, sex, and geographical area where they were rescued. Briefly, after admission to the Veterinary Teaching Hospital (VTH), fecal samples were collected from each animal following natural voiding. An aliquot of each sample was immediately stored at −20 °C and subsequently sent to the Laboratory of Infectious Diseases at the Department of Veterinary Sciences, where they were stored at −80 °C and subjected to nucleic acid extraction. Therefore, all molecular analyses on fecal samples were performed after the hospitalization period using stored frozen samples. Based on the results of molecular analyses conducted on the same panel of samples described in a previous scientific study [10], 23 samples that tested positive for at least one viral target in earlier research were selected for the present metagenomic investigation.

The sampling protocol and methods for the isolation of nucleic acids from fecal samples have been previously described in detail [10]. For the metagenomics analysis, DNA and RNA samples were pooled. Pools were assembled based on species and geographical recovery within municipalities in the Pisa province. Each pool contained a maximum of 3–4 nucleic acid samples, depending on availability. Samples were selected based on sample integrity and extraction yield to ensure a balanced representation of individuals while optimizing detection sensitivity.

### 2.2. Metagenomics Analysis

Six pools were assembled and subjected to NGS-based molecular investigations to characterize the fecal virome. Pools 1, 2, and 3 were made up of four fox (*Vulpes vulpes*) samples each. Pool 4 included samples from four badgers (*Meles meles*). Pool 5 consisted of samples from two badgers and one marten (*Martes martes*), while Pool 6 included samples from four porcupines (*Hystrix cristata*). To optimize detection, both DNA and RNA extracts were processed using sequence-independent single primer amplification (SISPA), which non-selectively amplifies viral genetic material [11,12,13,14].

The first step of the SISPA protocol consisted of RNA reverse transcription to synthesize single-stranded cDNA (ss-cDNA), using the SuperScript IV Reverse Transcriptase kit (Invitrogen—Thermo Fisher Scientific, Waltham, MA, USA) in combination with the semi-random primers FR26RV-N (5′-GCCGGAGCTCTGCAGATATC-N_6_-3′) and FR40RV-T (5′-GCCGGAGCTCTGCAGATATCTTTTTTTTTTTTTTTTTTTT-3′) (Eurofins Genomics, Ebersberg, Germany).

Subsequently, both the resulting cDNA and genomic DNA extracts underwent a first strand synthesis using DNA Polymerase I, Large (Klenow) Fragment (New England BioLabs, Ipswich, MA, USA), by adding 1 µL of NEBuffer and 1 µL of Klenow Polymerase to the previous reaction.

Finally, PCR amplification was performed using the universal primer FR20RV as the sole oligonucleotide, with the UCP HiFidelity PCR kit (Qiagen, Hilden, Germany).

Prior to sequencing, the SISPA-amplified products were quantified using a Qubit fluorometer to ensure adequate DNA concentrations for library construction. Negative extraction and amplification controls were included in each batch to monitor contamination. SISPA products, obtained from both RNA and DNA extractions (after Klenow preparation), were then combined in a 1:1 ratio before being sent to IGA Technology Services Srl (Udine, Italy). Sequencing libraries were prepared by IGA Technology Services following Illumina protocols, and final libraries were assessed for quality and fragment size using the Agilent Bioanalyzer (Agilent Technologies, Santa Clara, CA, USA) using 2100 Expert Software v.B.02.10). The shotgun sequencing was obtained using the Illumina^®^ NovaSeq 6000 platform (Illumina Inc., San Diego, CA, USA), producing 30 million paired-end 150 bp reads per sample. Raw sequence data underwent standard quality filtering and adapter trimming using FastQC (v0.11.9) and Trimmomatic (v0.39) before taxonomic analysis.

### 2.3. Bioinformatics Analysis

Initial taxonomic classification of sequences was performed by IGA Technology, the sequencing provider, using Kraken 2 software, a highly accurate and fast taxonomic classifier that uses exact k-mer matches to assign taxonomic labels to metagenomic sequences [15].

Additional bioinformatic analyses were performed using Geneious Prime^®^ 2022.2.2 (www.geneious.com) on a virtual machine provided by the University of Pisa’s Data Center (Windows 64-bit, dual Intel Xeon Gold 5120 CPUs at 2.20 GHz, 128 GB RAM).

**Figure 1:**
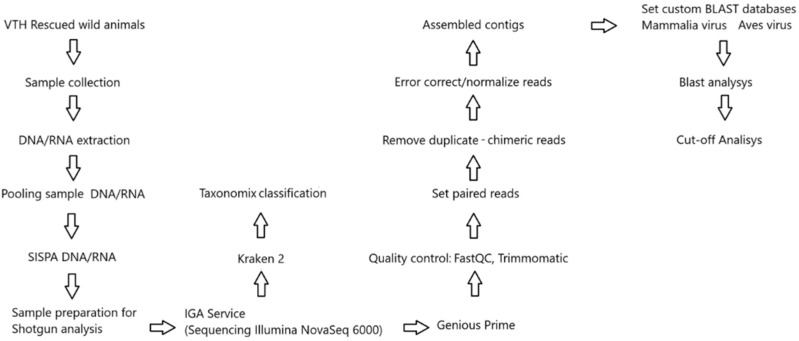
Metagenomic pipeline used for bioinformatic steps.

The analysis workflow applied to the fastq files generated by the sequencing service is illustrated in Figure 1.

Assembled “contigs” (from *de novo* assembly) were then subjected to BLAST analysis (BLAST+ v2.13.0) against two custom databases created from the NCBI Virus portal (https://www.ncbi.nlm.nih.gov/labs/virus/vssi/#/) access date 21 October 2022:

- Mammalia virus database:
  ○ Virus: viruses (taxid 10239)
  ○ Host: Mammalia (taxid 40674), excluding Homo sapiens (taxid 9606)

- Aves virus database:
  ○ Virus: viruses (taxid 10239)
  ○ Host: Aves (taxid 8782)

Databases included 470,053 viral sequences for mammals (1033 reference genomes) and 297,656 sequences for birds (212 reference genomes).

Sequences showing homology with database viruses were grouped by viral species. A representative reference sequence (GenBank accession number) was selected for each group. Sequence sets were mapped to the reference gene to generate a consensus sequence.

### 2.4. Bioinformatic and Statistical Parameters

The inclusion criteria applied for the positive identification of viral sequences were selected to ensure high confidence in the taxonomic classification and to reduce the risk of false positives, as recommended in viral metagenomic studies [16,17]. A consensus length >150 bp was set to exclude very short reads, which tend to yield low-confidence alignments and may match conserved motifs across unrelated taxa [18]. This threshold balances specificity and sensitivity, as shorter fragments are more likely to generate ambiguous hits or non-informative annotations [19].

Following BLAST analysis (BLAST: Basic Local Alignment Search Tool: https://blast.ncbi.nlm.nih.gov/Blast.cgi accessed on 24 July 2025), a minimum of >75% identical sites was required to ensure substantial sequence similarity across aligned regions, a criterion that improves the reliability of functional and taxonomic assignment, particularly when analyzing highly diverse viral populations [20]. Furthermore, a pairwise identity > 80% was used as a homology threshold, which is commonly employed in metagenomic pipelines to delineate true viral hits from noise and to avoid cross-mapping to conserved domains of non-viral origin [21].

E-value ≤ 10^−100^ represents a stringent statistical threshold for similarity searches using BLAST or similar tools. This value greatly minimizes the likelihood of random or low-confidence matches and is consistent with conservative cutoffs used in high-specificity pipelines [22,23]. Such stringency is particularly important in metagenomic analyses, where the complexity of environmental samples and incomplete viral databases can increase the risk of erroneous annotations [24].

Consensus sequences that failed to meet all these thresholds were excluded from downstream analysis to ensure that only sequences with strong homology and statistical support were retained for taxonomic classification and interpretation.

In detail, for each consensus, the following metrics were recorded:

- Length
- Number of identical sites
- Pairwise identity
- Reference sequence coverage
- E-value (vs. reference)

The inclusion criteria for positive identification were as follows:

- Consensus length > 150 bp
- Identical sites > 75%
- Pairwise identity > 80%
- E-value ≤ 10^−100^

## 3. Results

DNA quantification of the samples obtained following the SISPA protocol revealed concentrations ranging from 5 to 8 ng/µL. Based on these values, volumes between 12 and 16 µL per sample were submitted to ensure sufficient input for downstream analysis.

Shotgun metagenomic sequencing, performed on the Illumina^®^ platform, yielded between 1.90 × 10^7^ and 5.35 × 10^7^ reads per sample. The raw reads were initially subjected to taxonomic classification at IGA Technology using Kraken 2 [15]. For each sample, Kraken 2 analysis provided the number of total sequences, the proportion of classified and unclassified sequences, and their taxonomic assignments when available. As shown in Table 1, 80% to 97% of the total classified sequences were assigned to the domain Bacteria (taxid: 2). A smaller percentage, ranging from 0.4% to 1.7%, was identified as viruses (taxid: 10239) (Table 1).

To further identify the viral composition of the samples, an in-depth analysis was conducted using Geneious Prime^®^ 2022.2.2 (www.geneious.com), deployed on a virtual machine provided by the University of Pisa Data Center (Windows 64-bit OS, dual Intel Xeon Gold 5120 CPU 2.20 GHz, 128 GB RAM). This analysis involved comparison of the sequences against two custom databases built from the NCBI Virus platform (https://www.ncbi.nlm.nih.gov/labs/virus/vssi/#/ accessed on 24 July 2025), access date 21 october 2022. Sequences showing homology to viruses included in these databases were grouped by viral species, and a representative reference sequence was selected for each group, identified by its GenBank accession number (Table 1).

Of the 6 pools tested, 4 (38%) were positive for at least one viral target: 3/3 red fox pools (pool N° 1, 2 and 3), 1/2 mustelid pools (pool N° 5). Through metagenomic analyses, we identified eight distinct viral agents across diverse taxonomic groups: *Astroviridae*, *Circoviridae*, *Picornaviridae*, *Adenoviridae*, and *Retroviridae*. Among the six pools examined, four yielded positive hits for at least one viral target based on databases of viruses infecting Mammalia (*n* = 5) and Aves (*n* = 2). Within *Astroviridae*, sequences corresponding to both fox astrovirus and avian astrovirus were identified in Pool 1. *Pigeon circovirus* sequences were also detected in the same pool constituted by 4 fox samples. The *pigeon circovirus* scored as the highest percentage of coverage referred to reference sequence (MW656109) among all the other results. Within *Adenoviridae*, *Canine adenovirus* sequences were detected as the only viral match for Pool 3. We detected a member of the *Picornaviridae* family, namely *Tod virus 1,* resulting in 3 contigues for a total of 1492 bp with a coverage of reference sequence of 72.5% in Pool 2. Finally, two *Gammaretroviruses* were detected: *Murine leukemia virus* and *Rhinolophus ferrumequinum retrovirus*.

**Figure 2:**
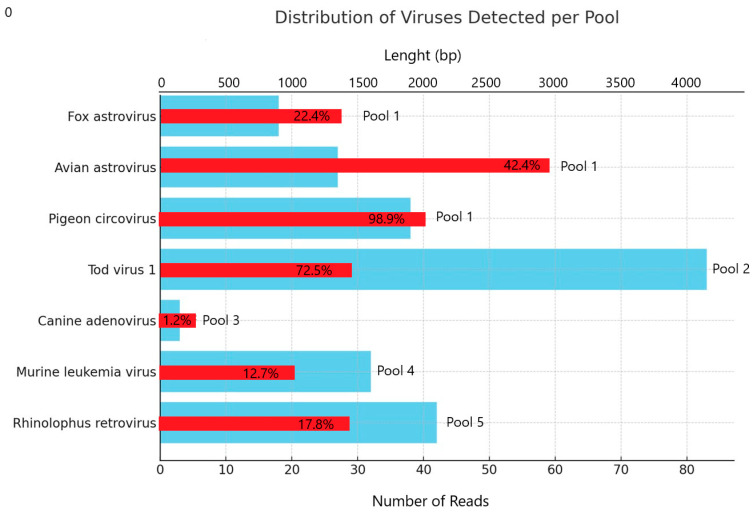
Bar chart showing number of reads assigned to each virus detected (light blue bar). The label next to the bar indicates sample poll number, and the red bar indicates the length of sequence and the reference sequence coverage percentage.

The corresponding GenBank accession numbers for the sequences generated in this study, along with their BLAST analysis results, are provided. For each sequence, the closest match available in public databases, the percentage of nucleotide identity, and the respective E-values are reported. The detailed results are summarized in Table 2 and Figure 2.

## 4. Discussion

The interface between domestic and wild animals represents a significant risk factor for pathogen transmission and provides a setting conducive to viral mutation and recombination, potentially enabling spillover into novel susceptible hosts. Since these animal groups often share pathogens and overlapping habitats—facilitating both direct and indirect contacts—areas where such interactions occur should be regarded as multi-host epidemiological systems deserving targeted health surveillance [25,26]. All samples analyzed in this study were obtained from wild animals rescued within the Tuscany region, specifically in the province of Pisa. In Tuscany, diverse and abundant wild mammal fauna are present, and much of Pisa’s landscape is dominated by urban-industrial or agricultural land uses. This configuration concentrates wildlife into restricted zones that frequently border human settlements and farms—creating an extensive domestic–wildlife interface. This situation often results in wild animals being injured in car collisions and, when feasible, being transported to the nearest veterinarian clinic for treatment. Despite increasing recognition of wildlife rehabilitation centers as valuable epidemiological sentinels, they remain underutilized for systematic pathogen surveillance. Indeed, few studies have leveraged these facilities either to monitor the health status of admitted animals or to systematically collect samples [27,28,29,30,31,32].

Fecal samples collected from rescued wild animals admitted to the VTH at the University of Pisa for clinical support were used in this investigation. We chose fecal matrices due to their widespread use in virological research, as evidenced by numerous published studies [9,33,34,35,36,37,38,39,40,41,42]. Several studies encompass both targeted investigations—employing classical molecular methods to detect specific pathogens within selected species [33,38,39,41,42]—and broader surveys aiming to characterize the fecal virome of wildlife using next-generation sequencing (NGS)-based metagenomics [9,34,35,36,37,41,43]. However, multispecies ecosystem-level virome surveys remain scarce. In this study, we analyzed pooled fecal samples grouped by species category as foxes, mustelids, and porcupines in a 12-month period. A limitation of this study is the relatively short sampling period, which may not capture long-term seasonal or anthropogenic influences on virome composition. On the other hand, the aim of this study is not to assess the impact of environmental and anthropogenic factors on the virome composition of wild animals, but rather to conduct a preliminary investigation that may lay the groundwork for future longitudinal evaluations in the same area or in regions affecting the same ecosystem. The fox is one of Europe’s most widespread wild mammals, with an omnivorous diet that includes carcasses and urban refuse. In recent years, fox populations have increased, particularly in peri-urban and urban settings [43,44,45]. Due to their ecological adaptability and behaviors, foxes are frequently regarded as sentinel species for ecosystem health assessments, spanning pollution, climate change, antibiotic resistance, and zoonotic disease monitoring [46,47,48,49,50,51]. We also included samples from species with scarce pathogen surveillance data, such as porcupines and mustelids, to enrich epidemiological knowledge of these wildlife groups. Fecal samples are widely used in viral surveillance of wildlife due to their high yield of viral nucleic acids and non-invasive collection method [9,34]. Compared to blood sampling, which requires restraint and can pose stress or risk to the animal, fecal sampling offers a practical alternative with proven effectiveness for detecting both enteric and systemic viruses shed in feces [35,36]. This approach is particularly suitable in rescue and rehabilitation settings, where animal welfare is paramount. This study confirms that fecal samples are suitable for molecular investigations, including advanced approaches such as shotgun metagenomic sequencing, in agreement with findings from previous research. Specifically, viral presence was detected in 4 out of 6 pooled samples: three pools (Numbers 1, 2, 3) originated from foxes, and one pooled sample (Number 5) consisted of two badgers and one marten. Regarding viral positivity, we were able to detect a total of 5 RNA viruses and 2 DNA viruses. Astroviruses are divided into *Mamastrovirus* (mammalian) and *Avastrovirus* (avian) lineages, the latter found in diverse domesticated and wild bird species [52,53,54]. Within *Astroviridae*, sequences corresponding to both fox astrovirus and avian astrovirus were identified. Notably, a longer astrovirus-like sequence was recovered from Pisa foxes via metagenomics compared to earlier PCR amplicons, improving alignment with fox astrovirus F5—a virus previously identified in the Netherlands but never reported in Italy [16]. The second sequence identified in this study showed high similarity to avian astroviruses from wild pigeons—two out of three contigs closely matched novel astroviruses recently recovered from Norwegian rock doves and woodpigeons [55]. These findings underscore the high genetic diversity among both *Avastroviruses* and *Mamastroviruses*, driven by interspecies transmission, including dietary crossover events. Moreover, the detection of avian astrovirus-like sequences in fox fecal samples may plausibly result from predatory or scavenging behavior, particularly in semi-urban ecosystems where high densities of pigeons coexist with wild carnivores such as red foxes. In these shared environments, dietary crossover through ingestion of infected avian prey likely contributes to the observed viral signatures. This is consistent with previous observations indicating that interspecies transmission, including predator–prey interactions, plays a key role in shaping astrovirus diversity and ecological distribution. Moreover, the persistence of synanthropic bird populations in peri-urban habitats may enhance the likelihood of such transmission events.

Regarding *Adenoviridae*, we detected *Canine adenovirus*, which aligns with our previous PCR findings confirming its circulation in the same geographic area [10]. Additionally, a member of the *Picornaviridae* family (*Tod virus 1*) was detected. Tod virus (a *Canine Picodicistrovirus*, CPDV) has been scarcely documented, and it is phylogenetically linked to insect-infecting *Dicistroviridae* [56,57]. Related viruses have also been identified in raccoons and hedgehogs [41,58], though the health implications of CPDV and related canine picornaviruses remain unknown. Within *Circoviridae*, we identified sequences matching *Pigeon circovirus* (PiCV), a predominant agent in young pigeons and the causative agent of young pigeon disease syndrome, often resulting in high mortality among juveniles [59,60]. PiCV is well-documented in North America, Australia, and parts of Europe, including Italy [60,61,62]. Our sequence, nearly full-length (~99% genome coverage), shared 94.5% nucleotide identity with strains from domestic pigeons. This result is consistent with previous findings, further supporting the hypothesis that fecal samples can reflect dietary-associated viral presence. This highlights the ecological value of fecal virome analysis for tracking indirect viral exposure pathways in wildlife.

Finally, we detected two *Gammaretroviruses*: *Murine leukemia virus* and *Rhinolophus ferrumequinum retrovirus*. *Gammaretroviruses* infect a broad spectrum of vertebrates, often causing leukemia, neurological disorders, and immunodeficiencies; their origins and their role in our sampled animals remain under investigation [63,64,65,66]. Our metagenomic data cannot distinguish whether these viral sequences represent exogenous, horizontally transmissible viruses or endogenous retroviral insertions. Therefore, caution is required when interpreting the biological relevance of retroviral sequences, particularly in the absence of supporting evidence such as viral replication markers or expression data. Additional analyses, including transcriptomic or integration site characterization, would be necessary to clarify their origin and potential role in host–virus interactions.

Overall, our metagenomic analyses produced results that diverged partially from prior PCR-based investigations on the same samples [10]. Some targets detected via PCR were not found in pooled metagenomic assays—likely due to dilution effects. Though PCR remains the gold standard for targeted pathogen detection, allowing nested amplification and high sensitivity, metagenomics offers broadened, unbiased coverage but introduces technical biases. While pooling strategies are useful for reducing costs and maximizing sample coverage, they may dilute low-abundance viral sequences, limiting detection sensitivity. Similarly, the sequence-independent single primer amplification (SISPA) method, while valuable for low-viral-load samples [67], can skew genomic coverage, underrepresent certain genomic regions, and inflate unclassifiable sequences. These limitations should be considered when interpreting metagenomic results and warrant caution in drawing conclusions about viral prevalence. Nevertheless, metagenomics uncovered pathogens that PCR would have neither targeted nor detected, especially those not previously associated with the sampled wildlife. Compared to previous PCR-based investigations [10], the metagenomic approach enabled the identification of viruses such as *Fox astrovirus F5* and *Pigeon circovirus*, which were not detected using targeted methods. This demonstrates how shotgun metagenomics can uncover previously undetected or unexpected viruses, particularly those not included in standard PCR panels. An advantage of metagenomics over traditional methods that we were able to directly observe is its ability to detect a broader range of viral taxa, including those not targeted by conventional PCR assays.

Our methodological approach enabled the detection of a wide spectrum of pathogens circulating in the studied ecosystems. Fecal sampling provided insights into pathogens not only in the sampled wildlife hosts but also in species predated upon or sharing the same habitats—consistent with carnivore and necrophage detection patterns. Next-generation molecular tools expanded virus discovery, confirming the role of wildlife as reservoirs and vectors of pathogens typically associated with domestic animals. We also identified viruses never reported in Italy or in the sampled species, emphasizing the current gap in our understanding of wildlife pathogen diversity. Although the viruses detected in this study were found in wildlife, several, such as avian astroviruses and pigeon circovirus, are well-documented in domestic poultry. The detection of these viruses in fecal samples of carnivorous mammals suggests dietary or environmental exposure, highlighting the potential for viral exchange at the wild–domestic interface. While we did not observe direct evidence of spillover to livestock, the shared environments between wild mammals, poultry farms, and ruminant pastures in Tuscany raise concerns about potential indirect transmission routes. Monitoring such a viral flow is essential to protect the health of livestock sectors, particularly under the One Health framework. None of the animals included in this study showed clinical signs indicative of systemic viral infections at the time of admission, suggesting that the detected viral sequences are likely to reflect subclinical shedding or passive carriage. These findings support the hypothesis that these wild mammals may act as asymptomatic reservoirs for certain viruses, rather than being clinically affected hosts. While correlation with clinical signs was limited in our dataset, future studies integrating clinical, pathological, and virological data will be essential to clarify pathogen–host dynamics. The detection of avian astroviruses and pigeon circoviruses in carnivorous mammals may reflect the dietary intake of infected birds. However, these viruses are known to cause disease in poultry, and their presence in urban wildlife raises concerns about potential environmental contamination and transmission risk to backyard flocks. Similarly, *Fox astrovirus*, although not clearly linked to disease, has been identified in multiple wild canid populations and could represent an under-recognized pathogen of veterinary relevance. Such findings underscore the need for integrated surveillance that considers both domestic animal and wildlife reservoirs.

These findings demonstrate that many viruses circulate silently, persisting subclinically, migrating across species boundaries, and evolving new host associations. Even viruses previously considered non-pathogenic warrant attention, as host–virus equilibrium, can shift under anthropogenic pressures. In an era marked by environmental disruption—climate change, globalization, pollution, habitat loss—these equilibria become precarious, increasing the likelihood of spillover events and outbreaks, even from seemingly innocuous viruses. Moreover, our findings contribute to a broader understanding of global zoonotic dynamics by demonstrating how subclinical viral circulation in urban/peri-urban wildlife may represent silent reservoirs for future spillover events. Viruses such as *Astroviruses* and *Circoviruses*, while often associated with mild or subclinical infections, may act as genetic reservoirs capable of recombination or host shifting, particularly in anthropized landscapes. These processes are central to the emergence of novel zoonotic threats. Therefore, sustained investment in infectious disease surveillance in wildlife is imperative. A One Health approach that integrates wildlife, domestic animals, and human health is essential to safeguarding ecological and public health resilience. This study confirms the utility of viral metagenomics in characterizing the fecal virome of wildlife in anthropized areas. The detection of potentially zoonotic or agriculturally relevant viruses underscores the importance of passive surveillance in rescue centers under a One Health framework.

## Figures and Tables

**Table 1:** Results of the taxonomic classification using Kraken 2.

Pool Number	N° of Sequences	Classified Sequences * (%)	Unclassified Sequences * (%)	Bacteria Sequences * (%)	Viral Sequences * (%)
1	2.54 × 10^7^	43	57	83	0.6
2	2.58 × 10^7^	29	71	86	1.7
3	2.03 × 10^7^	46	54	95	0.6
4	5.35 × 10^7^	89	11	97	1.3
5	3.91 × 10^7^	59	41	89	1.0
6	1.90 × 10^7^	48	52	80	0.4

The percentage of sequences assigned to Bacteria and Viruses is calculated over the number of classified sequences (*).

**Table 2:** Viral species identified in the analyzed pools.

Host	Virus	Ref-Seq	n° Reads	Length (bp)	Accession Number Contig > 150 bp	Ref-Seq Coverage	Identical Sites	Pairwise Identity	E Value
Pool 1 *Vulpes vulpes*	*Fox astrovirus*	KC692365.1	18	1443	PV999255 PV999256	1443/6456 22.4%	100%	100%	0.0
	*Avian astrovirus*	MF768270	27	2917	PV999257 PV999258 PV999259	2917/6872 42.4%	90.7%	96.1%	0.0
	*Pigeon circovirus*	MW656109	38	2015	PX067712	2015/2038 98.9%	92.4%	96.4%	0.0
Pool 2 *Vulpes vulpes*	*Tod virus* *(Canine* *picodicistrovirus)*	MT833880	83	1492	PV999260 PV999261 PV999262	1492/2058 72.5%	88.6%	97%	0.0
Pool 3 *Vulpes vulpes*	*Canine* *adenovirus*	Y07760.1	3	361	PX067713	361/30,536 1.2%	100%	100%	0.0
Pool 4 *Meles meles*	No significant viral sequences detected								
Pool 5 *Meles meles*	*Murine* *leukemiavirus*	KY574516	32	1039	PV999263 PV999264	1039/8191 12.7%	90.4%	93.1%	0.0
	*Rhinolophus* *ferrumequinum retrovirus*	JQ303225	42	1493	PV999265 PV999266 PV999267 PV999268	1493/8389 17.8%	98.4%	99.7%	0.0
Pool 6 *Hystrix cristata*	No significant viral sequences detected								

Results of Geneious and BLAST analyses of the obtained consensus sequences.

## Data Availability

The raw data supporting the conclusions of this article will be made available by the authors on request.

## Abbreviations

The following abbreviations are used in this manuscript:

VTH	Veterinary Teaching Hospital
SISPA	sequence-independent single primer amplification

## Appendix A

**Table A1:** Pool composition of wild mammal samples included in the study, For each sample, pool, sampling date, municipality of collection, sex, and cause of admission are reported.

	Pool	Sampling Date	Municipality	Sex	Admission
Fox	Pool 1	9 September 2020	Pisa	M	Intraspecific aggression
		28 October 2020	Vecchiano	M	Debilitation
		23 July 2021	Pontedera	M	Road accident
		30 July 2021	Pisa	M	Road accident
	Pool 2	2 March 2021	Ponsacco	F	Road accident
		1 March 2021	Pisa	F	Road accident
		25 August 2021	Capannoli	M	Road accident
		6 July 2021	Casciana Terme Lari	M	Debilitation
	Pool 3	23 October 2020	San Giuliano Terme	M	Road accident
		19 March 2021	Vecchiano	M	Road accident
		17 September 2021	Vecchiano	M	Road accident
		25 October 2021	Castelfranco di sotto	F	Road accident
Badger	Pool 4	10 November 2020	San Giuliano Terme	M	Road accident
		10 February 2021	Bientina	F	Unknown
		26 July 2021	Pisa	M	Road accident
		9 September 2021	Calci	M	Road accident
	Pool 5	22 March 2021	Fauglia	M	Road accident
		16 July 2021	Pisa	F	Intraspecific aggression
Marten		15 June 2021	Santa Luce	M	Debilitation
Porcupines	Pool 6	12 October 2020	Volterra	F	Intraspecific aggression
		23 March 2021	Bientina	M	Road accident
		16 July 2021	Pontedera	F	Road accident
		27 September 2021	San Miniato	M	Unknown
